# Molecular analysis of the *ABCA4* gene for reliable detection of allelic variations in Spanish patients: identification of 21 novel variants

**DOI:** 10.1136/bjo.2008.145193

**Published:** 2008-11-21

**Authors:** J Aguirre-Lamban, R Riveiro-Alvarez, S Maia-Lopes, D Cantalapiedra, E Vallespin, A Avila-Fernandez, C Villaverde-Montero, M J Trujillo-Tiebas, C Ramos, C Ayuso

**Affiliations:** 1 Genetics Department, Fundacion Jimenez Diaz, Madrid, Spain; 2 Centro de Investigacion Biomedica en Red de Enfermedades Raras (CIBERER), ISCIII, Madrid, Spain; 3 Visual Neuroscience Laboratory, IBILI, Faculty of Medicine, Coimbra, Portugal

## Abstract

**Background/aims:**

Mutations in *ABCA4* have been associated with autosomal recessive Stargardt disease (STGD), a few cases with autosomal recessive cone–rod dystrophy (arCRD) and autosomal recessive retinitis pigmentosa (arRP). The purpose of the study was threefold: to molecularly characterise families with no mutations or partially characterised families; to determine the specificity and sensitivity of the genotyping microarray; and to evaluate the efficiency of different methodologies.

**Methods:**

23 STGD, five arCRD and three arRP Spanish patients who were previously analysed with the ABCR400 microarray were re-evaluated. Results were confirmed by direct sequencing. In patients with either none or only one mutant allele, *ABCA4* was further analysed by denaturing high-performance liquid chromatography (dHPLC) and multiplex ligation-dependent probe amplification (MLPA). Haplotype analysis was also performed.

**Results:**

In the first analysis performed with the microarray, 27 *ABCA4* variants (27/62; 43.5%) were found. By dHPLC scanning, 12 novel mutations were additionally identified. In addition, two previously described mutations, one false negative (1/62; 1.6%) and one false positive (1.6%), were detected. MLPA analysis did not reveal additional substitutions. The new strategy yielded an increment of 21% compared with the approach used in the first round.

**Conclusion:**

*ABCA4* should be analysed by optimal combination of high-throughput screening techniques such as microarray, dHPLC and direct sequencing. To the best of our knowledge, this strategy yielded significant mutational spectrum identification in Spanish patients with *ABCA4*-associated phenotypes. Follow-up of patients, presenting an early onset of the disease and severe mutations, seems essential to perform accurate genotype–phenotype correlations and further characterisation of pathological *ABCA4* alleles.

Stargardt disease (STGD, MIM #248200) is the most common hereditary macular dystrophy, with an estimated prevalence of 1:10 000.^1^ It is characterised by onset in the juvenile to young adult years, decreased central vision, progressive bilateral atrophy of the retinal pigment epithelium (RPE) and the appearance of orange-yellow flecks around the macula and/or mid-periphery of the retina.^2^

The locus for recessive STGD was mapped to chromosome 1 (1p21–p13).^3^ Mutations in *ABCA4* have been described in autosomal recessive STGD (arSTGD),^4^ autosomal recessive retinitis pigmentosa (arRP),^5^ autosomal recessive cone–rod dystrophy (arCRD)^6^ and age-related macular degeneration (AMD).^7^

Up to now, ∼500 disease-causing mutations have been identified in *ABCA4*. The mutation spectrum ranges from single base substitutions to deletions of several exons, although the majority of reported changes are missense mutations. As has been described, mutations in this gene account for 66–80% of STGD-associated chromosomes.^8^ ^9^

Despite all the efforts of many research teams, there is a variable mutation detection rate, and this is often not satisfactory because there are still many undiscovered mutations. Therefore, the combination of different technical approaches, together with knowledge of the genetic background for a given population is important for the determination of novel mutations and for the genetic characterisation of these patients.

## METHODS

### Recruitment of subjects

A subset of Spanish STGD, arCRD and arRP patients previously tested for variants on the ABCR400 genotyping microarray described by Jaakson *et al*,^10^ in whom none or only one allele could be identified, was selected for further analysis. This molecular study was reviewed and approved by the Ethics Committee of the Hospital (Fundacion Jimenez Diaz) and adhered to the tenets of the Declaration of Helsinki (http://www.wma.org). Informed consent was obtained from all patients after the nature of procedures to be performed was fully explained.

### Clinical evaluation

Diagnoses of STGD, arCRD and arRP were determined according to a recessive mode of inheritance and were based on the following criteria:

- Diagnosis of STGD was determined according to a bilateral central vision loss with a beaten-bronze appearance and/or the presence of orange-yellow flecks in the retina from the posterior pole to the mid-periphery; typical dark choroid observed by fluorescein angiography; and normal to subnormal electroretinograms (ERGs).
- Diagnosis of CRD was based in initial complaints of blurred central vision without a history of night blindness, poor visual acuity, impairment of colour vision, funduscopic evidence of atrophic macular degeneration, peripheral disturbances including pigment clumping and/or pigment epithelial thinning, and greater or earlier loss of cone than rod ERG amplitude.
- RP was diagnosed in patients who developed night blindness early in life, peripheral vision loss, pigmentary retinal degeneration and markedly reduced scotopic ERG.

## MOLECULAR METHODS

### DNA extraction

Peripheral blood samples were taken, and genomic DNA was extracted using an automated DNA extractor (BioRobot EZ1, Qiagen, Hilden, Germany).

### Genotyping microarray

DNA samples from patients were analysed for variants on the ABCR400 microarray (http://www.asperbio.com), as described elsewhere.^10^ The 50 exons of the *ABCA4* gene, including the intron–exon junctions, were amplified using previously described PCR primers.^11^

### Direct sequencing

The sequencing reaction was performed with the Big-dye DNA Sequencing Kit (Applied Biosystems, Foster City, California). Sequence products were resolved in an ABIPrism 3130 (Applied Biosystems).

### Denaturing high-performance liquid chromatography

Denaturing high-performance liquid chromatography (dHPLC) sample screening was performed on a WAVE DNA Fragment Analysis System (Transgenomic). Because dHPLC does not usually differentiate the wild-type from the homozygous mutant sample, all unknown samples were analysed both singularly and mixed in a 1:1 proportion with a wild-type sample at the end of each PCR session and before heteroduplex formation.

### Multiplex ligation-dependent probe amplification

Multiplex ligation-dependent probe amplification (MLPA) reagents were obtained from MRC-Holland (SALSA MLPA kit P151-P152 ABCA4, Amsterdam), and the reactions were performed according to the manufacturer’s instructions (MRC-Holland).

### Fluorescent-PCR

In order to assess the size of one duplication found in exon 40 of the *ABCA4* gene, the PCR product was analysed in the ABIPrism 3130 (Applied Biosystems).

### Restriction assays

The presence of one novel missense mutation (p.Ile2047Asn) was analysed in 100 control chromosomes by conventional PCR amplification, restriction enzyme digestion (37°C overnight) with *TaqI* and analyses of the restriction fragments in a 3% agarose gel.

### Haplotype analysis

Haplotypes were constructed using three microsatellite markers flanking the *ABCA4* gene (TEL-D1S435-D1S2804-*ABCA4*-D1S236-CEN). Amplicons were electrophoresed using an ABIPrism 3130 (Applied Biosystems). For haplotype reconstruction, a computer program was used (Cyrillic version 2.1; http://www.cyrillicsoftware.com).

### Bioinformatics tool

In order to establish the nature of the variants located in the introns of the *ABCA4* gene, wild type and mutated sequences were analysed with Prediction Servers of Intron Splice Sites in human (Center for Biological Sequence Analysis of University of Denmark (http://www.cbs.dtu.dk/services)).

## RESULTS

### Molecular analysis

#### Further analysis of partially characterised families and identification of novel variants

**Figure 1:**
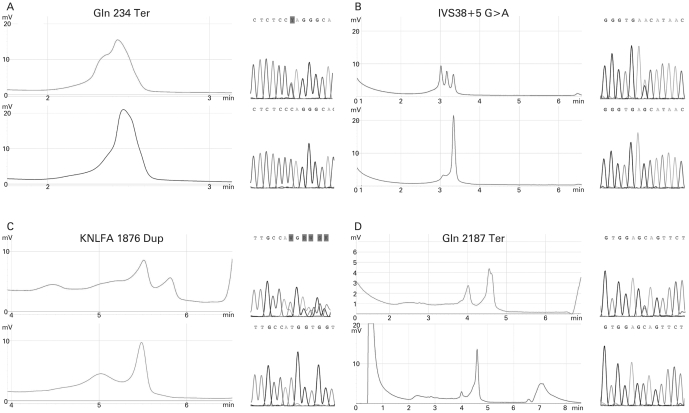
Chromatograms showing differences between the wild-type (down) and mutated profile (up). The wild-type sample shows a single peak, but the mutated sample shows different peaks. In some cases, the difference between chromatograms is very significant (B, C and D); in other cases, it is not so (A). Nevertheless, these samples were confirmed by sequencing for all family members.

A total of 31 Spanish patients, who were previously tested for mutations with the ABCR400 microarray, were further analysed with two different techniques: dHPLC and MLPA. In 27 out of 31 patients, only one disease-associated allele was detected using the genotyping microarray, and in the remaining cases, no variant was detected by the chip (four out of 31 patients). Therefore, the mutation detection rate for the microarray was 43.5%. However, a subsequent analysis performed by dHPLC (fig 1) led to the identification of 24 sequence variations in the *ABCA4* gene (tables 1, 2).

**Table 1:** Clinical findings of the Spanish patients with Stargardt disease (STGD), autosomal recessive cone–rod dystrophy and autosomal recessive retinitis pigmentosa

Pedigree	Age (years)	Age (years) of onset	Visual acuity		Diagnosis	Allele 1		Allele 2		Segregation
			OD	OS		Nucleotide changes (exons)	Amino acid change	Nucleotide changes (exons)	Amino acid change	
ARDM-135	42	24	0.4	0.6	STGD	c.5882G>A(42)	p.Gly1961Glu	c.1029_1030insT**(8)**	p.Asn344fsX	**NP**
ARDM-240	15	13	0.2	0.16	STGD	c.5882G>A(42)	p.Gly1961Glu	**c.2285C>A**(15)	**p.Ala762Glu**	Yes
ARDM-225	32	25	0.25	0.50	STGD	c.5882G>A(42)	p.Gly1961Glu	**c.6559C>T**(48)	**p.Gln2187X**	Yes
ARDM-164	21	11	NA		STGD	c.3386G>T(23)	p.Arg1129Leu	c.700C>T**(6)**	p.Gln234X	**Yes**
ARDM-162	50	16	0.1	0.1	STGD	c.3386G>T(23)	p.Arg1129Leu	ND	ND	Yes
ARDM-198	27	19	0.1	0.1	STGD	c.3386G>T(23)	p.Arg1129Leu	ND	ND	NP
ARDM-125	31	9	0.3	0.4	STGD	c.3211insGT(22)	FS		**p.KNLFA1876dup**	Yes
ARDM-158	24	9	0.2	0.2	STGD	c.3211insGT(22)	FS	c.4537delC**(30)**	p.Gln1513fsX1525	**NP**
ARDM-165	40	30	NA		STGD	c.3211insGT(22)	FS	ND	ND	NP
ARDM-167	49	23	0.05	0.05	STGD	c.3211insGT(22)	FS	ND	ND	NP
ARDM-146	32	13	0.06	0.1	STGD	c.3056C>T(21)	p.Thr1019Met	**c.6140T>A**(44)	**p.Ile2047Asn**	Yes
ARDM-40	46	9	0.1	0.1	STGD	c.3056C>T(21)	p.Thr1019Met	**c.3943C>T**(27)	**p.Gln1315X**	Yes
ARDM-90	26	8	Hand moving		STGD	c.5929G>A (43)	p.Gly1977Ser	**IVS21-2A>T**		Yes
ARDM-181	57	16	0.1	0.09	STGD	c.3323G>A (22)	p.Arg1108His	**IVS38+5G>A**		Yes
ARDM-197	35	15	0.1	0.1	STGD	c.4793C>A(34) (false +)	p.Ala1598Asp (false +)	**c.5172G>T**(36)	**p.Trp1724Cys**	Yes
ARDM-183	63	55	0.150	0.175	STGD	c.6079C>T(44)	p.Leu2027Phe	**c.5929G>A**(43) (false –)	**p.Gly1977Ser** (false –)	NP
ARDM-38	35	6	0.01	0.02	STGD	c.1804C>T(13)	p.Arg602Trp	**c.4739delT**(33)	**p.Leu1580fs**	Yes
ARDM-163	48	32	0.01	0.32	STGD	c.4457C>T(30)	p.Pro1486Leu	ND	ND	Yes
ARDM-166	42	39	NA		STGD	c.6320G>A(46)	p.Arg2107His	ND	ND	Yes
ARDM-222	26	23	NA		STGD	c.2791G>A(19)	p.Val931Met	ND	ND	NP
ARDM-160	30	5	0.25	0.1	STGD	ND	ND	ND	ND	Yes
ARDM-173	49	7	NA		STGD	ND	ND	ND	ND	Yes
ARDM-205	NA	NA	NA		STGD	c.4919G>A(35)	p.Arg1640Gln	ND	ND	NP
ARDM-247	30	12	0.05	0.1	CRD	c.3386G>T(23)	p.Arg1129Leu	**c.6410G>A**(47)	**p.Cys2137Tyr**	Yes
ARDM-99	59	46	0.05	0.05	CRD	c.4297G>A(29)	p.Val1433Ile	ND	ND	NP
ARDM-131	27	15	0.9	0.7	CRD	c.2701A>G(18)	p.Thr901Ala	ND	ND	Yes
ARDM-100	28	4	0.2	0.16	CRD	ND	ND	ND	ND	Yes
ARDM-142	30	25	0.8	0.5	CRD	ND	ND	ND	ND	Yes
RP-773	38	20	0.05	0.05	RP	c.33N86G>T(23)	p.Arg1129Leu	ND	ND	NP
RP-959	53	10	0.1	0.1	RP	c.466A>G(5)	p.Ile156Val	ND	ND	Yes
RP-1058	37	6	0.2	0.6	RP	c.4297G>A(29)	p.Val1433Ile	ND	ND	NP

Twenty-seven out of 31 subjects were found to be compound heterozygous for mutations in the *ABCA4* gene detected by microarray. These mutations were confirmed by the denaturing high-performance liquid chromatography (dHPLC) technique and sequencing analysis. In patients from families ARDM-135, ARDM-240, ARDM-225, ARDM-164, ARDM-125, ARDM-158, ARDM-146, ARDM-40, ARDM-90, ARDM-181, ARDM-197, ARDM-183, ARDM-38 and ARDM-247, the second mutation was found by dHPLC (in bold).

FS, frameshift; NA, not available; ND, not detected; NP, not performed; OD, right eye; OS, left eye.

**Table 2:** Synonymous and non-synonymous codon variants and intronic variants in the ABCA4 gene

Exon	Nucleotide change	Sequence change	Reference
3	**c.264 A>T**	**p.Gly88Gly**	This study
	**IVS2-27G>A**		This study
	IVS3+26 A>G		9,15,18
	IVS6-32 T>C		9,15
8	**c.1029T>C**	**p.Asn343Asn**	This study
10	**c.1299A>G**	**p.Glu433Glu**	This study
12	**c.1654G>A**	**p.Val552Ile**	This study
	**IVS13+16G>A**		This study
19	**c.2832A>G**	**p.Pro944Pro**	This study
20	c.2964C>T	p.Leu988Leu	18
23	**c.3507G>A**	**p.Gln1169Gln**	This study
	IVS28+43G>A		24
	IVS43-16G>A		25
	**IVS48+39 T>A**		This study
	IVS48-3T>C		15,18

Novel changes are shown in bold.

**Figure 2:**
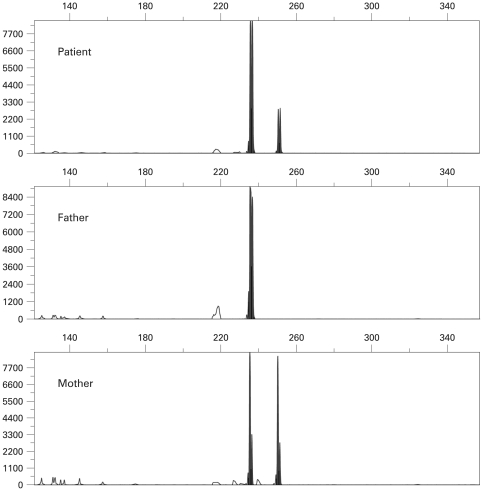
Electropherogram showing the five-amino-acid duplication (p.KNLFA1876dup) in the patient from family ARDM-125. By this design, we could confirm that this gain was of maternal origin.

Eight of these changes were interpreted as null mutations: three were nonsense mutations (p.Gln234X, p.Gln1315X “de novo” and p.Gln2187X); two splice-site mutations (IVS22-2A>T and IVS38+5G>A); three frameshift variants: two caused by the insertion or deletion of one nucleotide (c.1029_1030insT and c.4739delT) and one duplication of five amino acids (p.KNLFA1876dup). We were able to determine that this duplication was of maternal inheritance (fig 2). Three missense changes were detected (p.Ala762Glu, p.Ile2047Asn and p.Cys2137Tyr) in patients but not in the 100 ethnically matched control chromosomes. We identified the heterozygous p.Ile2142Val change in one out of 100 control chromosomes. Interestingly, this variant has not been previously described and was not found in Spanish STGD/CRD patients. Moreover, two previously described mutations [c.4537delC and p.Trp1724Cys], not included in the array by the time of the screening, and one false negative [p.Gly1977Ser] were detected (1/62; 1.6%).

Despite these putative disease-associated alleles, additional variants interpreted as non-pathogenic were identified. Additionally, one novel change in exon 12 (p.Val552Ile) was found in one STGD patient by means of the dHPLC technique. This variant was detected in two alleles out of 200, suggesting a polymorphism. We also found six variants that did not produce a change at the protein level, five being novel ones and eight intronic variants, three of them previously unreported (table 2). These three changes located in the introns were analysed with a bioinformatics tool. According to this analysis, these variants are not predicted to affect the splicing process.

Finally, in 17 of 31 patients in whom the second mutant allele or no disease-associated alleles were found, we assessed dosage for the *ABCA4* locus using the MLPA technique. No deletions or duplications were found for any of the 48 amplified probes in these cases. These results were compared with two control samples.

Despite the high allelic heterogeneity and the large amount of novel *ABCA4* variants detected in this screening, the p.Arg1129Leu mutation was found to be the most frequent disease-associated allele (5/62; 8%).

**Figure 3:**
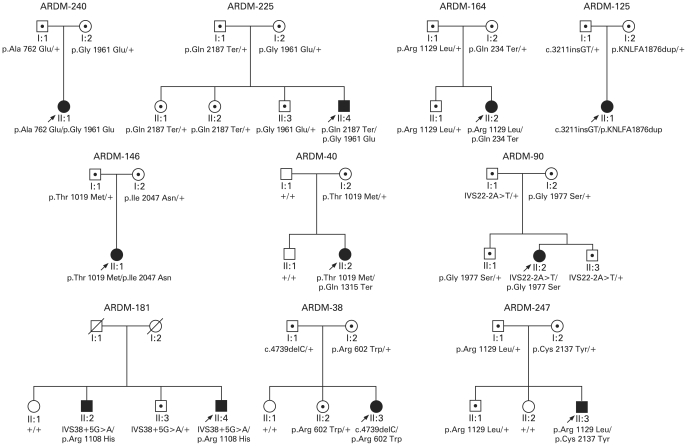
Family pedigrees, all diagnosed as having Stargardt disease except for family ARDM-247 whose proband presents a cone–rod dystrophy phenotype. The family number is shown above each pedigree. Probands are indicated with an arrow.

Segregation analyses were performed in all families in which samples from additional family members were available. In 11 STGD families and one CRD family, where an additional pathogenic change was found, correct segregation of the disease alleles was demonstrated (fig 3). Haplotype analysis was also performed, and cosegregation was observed (available on request).

The combination of direct sequencing, together with dHPLC and MLPA techniques allowed us to further analyse the *ABCA4* gene in STGD, arCRD and arRP cases, therefore leading to the identification of 21 novel sequence variants. In addition, the specificity, sensitivity and efficiency of different methodologies were evaluated.

#### Determination of sensitivity and specificity of the ABCR400 microarray

We identified 1.6% (1/62) of false positives and 1.6% (1/62) of false negatives for the ABCR400 microarray. In the patient belonging to family ARDM-197, the chip detected the p.Ala1598Asp mutation. However, dHPLC and direct sequencing demonstrated that this mutation was not present (false positive). Interestingly, the p.Trp1724Cys mutation was detected by dHPLC in this family. In contrast, the dHPLC technique led to the detection of the p.Gly1977Ser mutation in the patient from the family ARDM-183, but the chip did not detect it (false negative). Therefore, the values of sensitivity and specificity of the genotyping microarray were 96.3% and 97.1%, respectively.

#### Evaluation of the efficiency of different methodologies

The genotyping microarray identified a total of 27 disease-associated alleles, yielding a mutation detection rate of 43.5%. As we currently confirm these results by sequencing, a false positive [p.Ala1598Asp] was identified. Altogether, the combination of several high-throughput approaches—ABCR400 microarray, dHPLC, MLPA and direct sequencing as a confirmation method—led to the detection of 12 novel mutations and one false negative. Therefore, the detection rate was increased by 21%, mainly due to dHPLC scanning, achieving a mutation detection rate of 64.5% (40/62). Moreover, two previously described pathogenic variants were also detected. In addition, nine novel polymorphisms were identified.

### Genotype–phenotype correlation

#### STGD patients presenting one putative null allele

Importantly, putative null mutations—including frameshifts, nonsense, or splice-site variants—were observed more frequently (7/31 patients; 22.6%) in compound heterozygous alleles from patients with age of onset of the disease <10 years, all of them presenting a STGD phenotype (table 1). A total of 13 putative null disease-associated alleles (13/62; 20.9%) were identified in this study. The majority of patients who presented at least one putative null mutation presented early-onset symptoms, except for two STGD patients belonging to the families ARDM-135 and ARDM-225, whose disease started at the age of 24–25 years. Moreover, both patients presented the p.Gly1961Glu mutation, which is considered an allele of moderate effect.^7^

#### STGD patients presenting two putative null alleles

Patients from families ARDM-125 and ARDM-158 were carrying two putative null *ABCA4* alleles (table 1). Both of them harboured the previously reported c.3211insGT mutation. For the second disease-associated allele, the patient from family ARDM-125 showed a duplication of five amino acids (p.KNLFA1876dup), while the patient from family ARDM-158 presented the frameshift c.4537delC mutation. Probands from these families presented a STGD phenotype, presenting symptoms since the age of 9.

#### Patients presenting arCRD and arRP phenotypes

Interestingly, in our patients with arCRD and arRP phenotypes, no putative null mutations were found (table 1). In three arRP cases, the second disease-associated allele was not identified. In one of them, cosegregation of the mutation within the family was observed.

## DISCUSSION

Thirty-one Spanish families were analysed for mutations in *ABCA4*. This gene encodes the *ABCA4* protein, a member of the ATP-binding cassette (ABC) transport super-family. It is involved in the transport of vitamin A derivates across the membrane of the outer segment discs of photoreceptors.^12^ ^13^

The frequency of mutated alleles found with the microarray was 43.5% (27/62; 33.8% (21/62) for STGD patients, 4.8% (3/62) for arCRD patients and 4.8% (3/62) for arRP patients). However, the frequency of mutated alleles detected with microarray and dHPLC was 54.8% (34/62) for STGD patients and 6.4% (4/62) for arCRD patients. In contrast, the mutation detection rate for arRP cases was not increased by dHPLC scanning, as no additional disease-associated alleles were found.

The p.Arg1129Leu mutation was found to be the most frequent missense variant, representing 8% (5/62) of the total pathogenic alleles (table 1). In previous studies of the Spanish population, the p.Arg1129Leu variant was identified as a major mutant allele which accounted for 24% of the STGD alleles.^14^ This variant has been postulated to have a moderately severe effect and has predominantly been associated with a STGD phenotype.^14^ In contrast, the prevalence of this mutation in patients from North America was less than 1%.^15^ Interestingly, we identified one 30-year-old patient (ARDM-247), double heterozygous for the p.Arg1129Leu and p.Cys2137Tyr alleles, who presented a CRD phenotype. This p.Cys2137Tyr change was located more towards the amino terminus. Moreover, in other study, the results showed that the changes located in this zone appear to result in altered processing of the protein and to be associated with an earlier onset of disease.^16^ The p.Cys2137Tyr change in combination with the p.Arg1129Leu allele produced a CRD phenotype. Therefore, we speculate that the novel p.Cys2137Tyr variant could be a severe allele which is modifying the patient’s phenotype.

The second most prevalent missense disease-associated allele was p.Gly1961Glu (3/62; 4.8%), presenting a lower frequency than other European populations.^10^ This mutation was found in three STGD patients, two of them presenting at least one putative null mutation (c.1030insT and p.Gln2187X), who presented symptoms at the age of 24–25 years. Thus, we could suspect a moderate effect for the p.Gly1961Glu allele, as previously reported.^7^

In relation to null alleles, the most frequent mutation was c.3211insGT, identified in four independent STGD families out of 31 (6.4%). In two of these cohorts (ARDM-125 and ARDM-158), the second mutated allele was found by dHPLC (p.KNLFA1876dup and c.4537delC, respectively). In both patients, first symptoms of the disease appeared at the age of 9 years old. Nevertheless, in patients from families ARDM-165 and ARDM-167, in whom the second disease-associated allele was not detected, the age of onset of the disease was at the third decade of life (table 1). Therefore, we speculate that the second—not yet detected—mutation might be of a moderate effect, as the disease started later in life. The c.3211insGT allele has been previously described in other studies.^17^^–^19. Rozet *et al*^17^ reported two STGD patients who were compound heterozygous for the c.3211insGT allele and another sequence change, whose age of onset was before 12 years old.^17^ In other population study performed by Briggs *et al*,^18^ two STGD patients harbouring the heterozygous c.3211insGT allele were described. These probands presented reduced central visual acuity before the age of 30. Interestingly, Paloma *et al*^19^ described one CRD case, compound heterozygous for the c.3211insGT and p.Arg212Cys variants, showing symptoms at the age of 9. Considering these previously reported genotype–phenotype correlations, together with the genetic and clinical findings described in our Spanish patients, we could hypothesise that the c.3211insGT variant is predominantly associated with STGD, while the second mutated allele might have a modulating effect on the patient’s phenotype.

Two additional null alleles, associated with STGD, were identified. The patient from family ARDM-125 presented the novel p.KNLFA1876dup, located at the second transmembrane region of the *ABCR* protein, while the patient from family ARDM-158 harboured the frameshift c.4537delC variant that causes a premature stop codon 12 amino acids downstream.

Nevertheless, as the c.3211inGT, p.KNLFA1876dup and c.4537delC are null alleles, and some patients presented symptoms early in life (9 years), we cannot rule out that they could develop a more severe phenotype (arCRD) in the future. Indeed, a similar situation has been previously reported in one Spanish patient homozygous for the c.2888delG allele,^20^ whose clinical phenotype changed from STGD (age of onset 10 years) to arCRD (age of diagnosis 26 years), during the course of the study.

Two novel splice-site alterations were described, IVS21-2A>T (ARDM-90) and IVS38+5G>A (ARDM-181). Both patients presented STGD phenotype, and the age of onset of the disease corresponded to the first and second decade of life, respectively. In these cases, the total inactivation of the splice site is unlikely, although these changes could alter the splicing of corresponding exon, creating cryptic sites, thus producing a longer protein.^21^

In two patients diagnosed as having arCRD and arRP phenotypes respectively, we identified the missense p.Val1433Ile mutation, located in the second transmembrane domain. This sequence change has been previously associated with age-related macular degeneration (AMD)^7^ and has also been reported in one arCRD patient, although it was questioned whether the nature of this amino acid change was pathological or not.^22^ We investigated this change further in the Spanish control population, and it was not found in 124 ethnically matched chromosomes, thus suggesting a pathogenic effect.

Family RP-959 was previously analysed by the genotyping microarray, and the p.Ile156Val allele was detected.^20^ This variant has been associated with the STGD phenotype. In the affected individual, the second mutated allele was not found by dHPLC. Segregation analysis of the mutation within the family did not allow this gene to be excluded. Thus, the presence of RP phenotype and this mutation could be explained because the second mutation would be a severe allele or would be due to fortuitous association. Moreover, in the RP-773 and RP-1058 families, it was not possible to detect the second mutated allele, so the implication of mutations in this gene in the development of RP is still not clear as was previously reported.^20^

To establish the nature of the three non-reported changes located in the introns, we used a bioinformatics tool to analyse and compare the wild type and mutated sequences. The results demonstrated that these variants probably did not modify the splicing process.

By MLPA analysis, no deletions or duplications were found in patients. Indeed, it has been suggested that these alterations contribute to only a small fraction of disease-associated alleles for *ABCA4*.^23^

The genotyping microarray is a comprehensive screening tool for genetic variation in patients with *ABCA4*-associated retinal pathology with a high reliability. Despite the relative small number of families, a huge number of *ABCA4* disease-associated alleles were identified, representing an increment of 4% of novel variants described in this gene, as ∼500 sequence changes have been described so far. To the best of our knowledge, this optimal screening strategy represents extensive mutational spectrum identification in Spanish patients with *ABCA4*-associated phenotypes.

Moreover, these new mutations could be added to the new versions of the ABCR400 microarray, thus increasing the detection and validity of the array. The combination of these three different techniques of screening (microarray, dHPLC and MLPA) allowed us to reach a complete diagnosis of 14 of 31 patients with only one mutant allele identified, because of the detection of the second disease-associated allele with by dHPLC. In addition, it is probable that a great number of mutations might reside in parts of the gene (eg, the promoter region or the introns) that have not yet been identified.

Given an estimated prevalence of STGD (1:10 000), the Hardy–Weinberg equilibrium would indicate that the heterozygous state can be expected in about 1/50.^24^ So many variants, such as p.Val552Ile, could be really a mutation found in normal population. Thus, expression analysis could be an interesting tool with which to discriminate the pathological effect of these changes.

Screening of increasingly large numbers of patients from distinct populations would help to determine whether this broad spectrum of mutations can be explained by ethnic differences or is an indicator of extensive allelic heterogeneity of *ABCA4* in Stargardt disease and other retinal diseases. Follow-up of patients, especially those presenting an early onset of the disease and harbouring severe *ABCA4* mutations, would help in performing accurate genotype–phenotype correlations and in further characterisation of pathological alleles.
